# Rise in cesarean section rate over a 20-year period in a public sector hospital in northern Thailand

**DOI:** 10.1007/s00404-012-2531-z

**Published:** 2012-08-30

**Authors:** Chitrakan Charoenboon, Kasemsri Srisupundit, Theera Tongsong

**Affiliations:** Department of Obstetrics and Gynecology, Faculty of Medicine, Chiang Mai University, Chiang Mai, Thailand

**Keywords:** Cephalopelvic disproportion, Cesarean section, Cesarean section rate, Pregnancy

## Abstract

**Objective:**

To determine a trend of cesarean section rate (CSR) and main contributing factors in a public sector hospital, representing northern part of Thailand.

**Methods:**

A retrospective descriptive analysis was conducted by assessing the database of maternal-fetal medicine unit, which had prospectively been collected for 20 years. Trends were evaluated using data for the years 1992–2011. Private sector patients were excluded.

**Results:**

A total of 50,872 public sector patients were available for analysis. The number of deliveries was gradually decreased from 3,802 in 1992 to 1,748 in 2011. Of them, 7,480 underwent cesarean section, CSR of 14.7 %. However, the CSR was significantly increased from 11.3 % in 1992 to 23.6 % in 2011 (*p* value <0.001). The CSRs indicated by cephalopelvic disproportion (CPD) and previous CSs were mainly responsible for a marked increase over the study period. CSR due to CPD was increased from 3.2 % in 1992 to 7.9 % in 2011 (*p* value <0.0001). While CSR due to other indications either breech presentation, fetal distress and twin pregnancies were only slightly, but significantly increased in the last decades but they are relatively constant in the recent years.

**Conclusions:**

In our public sector, CSR has gradually increased. The main reasons of such an increase were likely to be associated with over-diagnosis of CPD and subsequent repeated CS, while other indications played only a minimal role. To achieve the appropriate CSR, audit system for diagnosis of CPD must be instituted.

## Introduction

Cesarean section (CS) with appropriate indications is an essential surgical procedure in modern obstetrics, which can certainly save the lives of mothers and fetuses. Nevertheless, a large number of CSs are currently performed without medical necessity and are associated with higher risks than benefits. CS is the most common operation, taking away resources from medically necessary care. According to WHO recommendations, a CS rate (CSR) between 5 and 15 % of total births is an optimal rate resulting in high efficacy [1–4]. Based on the best evidence to date, a frequency of between 5 and 10 % seems to achieve the best outcomes. The cesarean section of more than 15 % is not associated with reductions in maternal and neonatal mortality and morbidity [4], but rather unnecessary, inappropriate, and riskier than beneficial in term of better health outcomes [5, 6]. A substantial proportion of cesarean sections are associated with maternal and fetal risk without medical benefit, so-called unnecessary CS [2, 7]. A WHO study showed that cesarean section without clear indication has an increased risk of 280 % for severe adverse short-term outcomes for the mother as compared to spontaneous vaginal delivery (42/1,000 compared to 15/1,000, respectively) and nearly six times as much if before labor onset, but after labor onset 14 times as much [8]. During the last three decades, the CSR has been significantly increased in both developed and developing countries. Globally, the overall CSR has risen from <7 % in the 1970s to over 25 % in 2003, with an even greater increase in poor countries [9–11].

The rise in CSR is associated with numerous factors. Private service is one of the well-established factors causing an increasing CSR [2, 12]. In addition, it has been thought that house staff in public institutions may be held more strictly to conservative protocols than the private physicians. Even in public hospitals the CSR is also too high. Several non-incentive factors can lead to a higher CSR, for examples: improved surgical techniques reducing rate of operative complications [13], demographic factors such as increased maternal age at pregnancy and higher frequency of repeat CS [14], physician’s attitudes towards CS [15], and request from mothers who may believe that CS protects against urinary incontinence and prolapsed [16]. Unnecessary CSs are one of the most concerns in modern obstetrics. To achieve the optimal CSR and avoid morbidity and mortality related to unnecessary CS, underlying causes of the increased CSR must be first analyzed. Though several studies have been published on trends of CSR, CSRs are markedly different from study to study. In some countries the true CSR is far higher than the recommended range, for example, the CSR in public hospitals in Tehran, Iran, has risen from 14.3 % in 1979 to 85.3 in 2009 [17]. Moreover, most studies did not determine the main indications contributing to a higher rate.

To identify the extent of unnecessary CSR in our setting and as to what indications mainly contributed to a higher rate, we therefore, performed this study to determine the trend of CSR in a public hospital in Thailand, not including private services, during the past two decades in order to provide an actual national trend and guideline to develop policy.

## Materials and methods

**Fig. 1:**
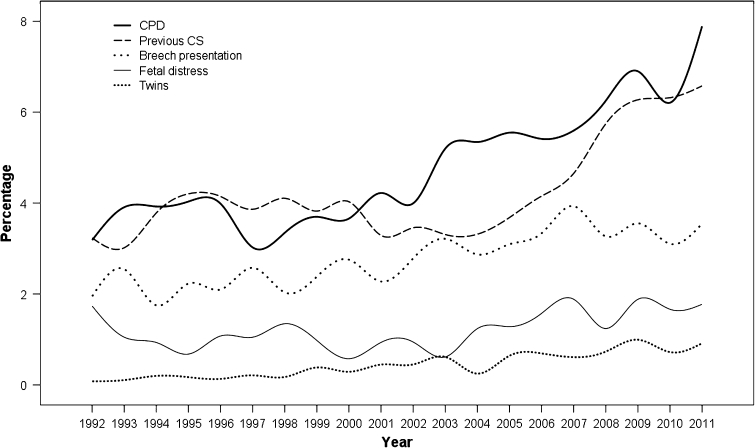
Graph depicts CSR trends during 20 year period according to main indications

A descriptive analytic study was conducted at Maharaj Nakorn Chiang Mai Hospital (public hospital), Chiang Mai University, a tertiary center as well as a teaching hospital in the northern part of Thailand, serving mainly local population with mixed socioeconomic status. Decision on cesarean section was made by the staffs, certified obstetricians, in charge, though most patients were primarily taken care by the residents. This study was ethically approved by the local institutional review board. The database of maternal-fetal medicine unit was assessed and reviewed for baseline characteristics, and labor outcomes. Our maternal-fetal medicine database is a prospective database, which has been established since 1989. The database contains a structured checklist collecting data from medical records pertaining to all deliveries in the hospital. The date of delivery, maternal age, parity, gestational age at delivery, place of residence, mode of delivery (vaginal or cesarean section), as well as, indications for operative procedures, obstetric or medical complications, and person in charge of delivery (medical staffs, residents, medical students, or staff nurses) and etc, were included in the collected information. These patient’s data have been recorded on daily basis. In the present study, the data was gathered during the past two decades (1992–2011). Inclusion criteria were all pregnant women, who gave birth at Maharaj Nakorn Chiang Mai Hospital at gestational age of 24 weeks of gestation or more, including both singleton and multifetal pregnancies. Exclusion criteria were deliveries taken care as private practice. Main outcomes measures included CSR per year, changes in CSR during the 20 year period of study, and percentages of various indications for cesarean sections each year (Fig 1).

### Statistical analysis

Data were analyzed using the SPSS version 17.0 software package (SPSS, Inc., Chicago, IL). Means and standard deviation for continuous variables and percentage or proportions for categorical data were calculated. Percentages of cesarean rate per years due to various indications were determined. Chi square and correlation were used to test for significance of CSR changes over the period of study. *p* < 0.05 was considered statistically significant.

## Result

During the study period, 72,800 deliveries were reviewed. Of them, 21,928 were private cases and were excluded. The remaining 50,872 were available for analysis, including 27,380 nullipara and 23,492 parous women. The number of deliveries among public sector patients was gradually decreased from 3,802 in 1992 to 1,748 in 2011. Of them, 7,480 underwent cesarean section, CSR of 14.7 %. CSR in primipara and mulitpara was 13.4–16.2 %, respectively (*p* value <0.001). The CSR was significantly increased from 11.3 % in 1992 to 23.6 % in 2011 (*p* value <0.001), as seen in Table 1. The percentage distribution of indications for CS in each year is presented in Table 2 and Fig. 1. CSR indicated by failure of labor induction, fetal malpresentation, placenta previa, prolapsed cord and HIV antibody positive were not increased significantly over the study period. Notably, rates of CS due to breech presentation, multifetal pregnancies and fetal distress were slightly but significantly increased but they were relatively constant during the last decade. CSRs indicated by CPD and previous CS were mainly responsible for marked increase over the study period. CSR due to CPD was increased from 3.2 % in 1992 to 7.9 % in 2011 (*p* value <0.001). Likewise CSR due to previous CS was increased from 3.2 % in 1992 to 6.6 % in 2011 (*p* value < 0.0001). Means maternal age was slightly, but significantly increased from 26.79 ± 5.5 years in 1992 to 27.88 ± 6.0 years in 2011 (*p* value <0.001). The proportion of elderly pregnancies (≥35 years) was markedly increased from 9.6 % in 1992 to 15.0 % in 2011. However, teen-age pregnancies was rather unchanged, 8.9–9.1 % (*p* = 0.06). Additionally, mean gestational age (37.7 ± 12.1 weeks), and birth weight (2,866 ± 669 g) were relatively unchanged during the study periods.

**Table 1:** Annual total births and cesarean section rate (%) over the past two decades

Years	Number of deliveries	Cesarean section (%)
1992	3,802	11.3
1993	3,812	11.8
1994	3,542	11.9
1995	3,545	12.7
1996	3,812	13.2
1997	3,339	12.0
1998	2,899	12.9
1999	2,353	13.1
2000	2,432	13.2
2001	2,463	13.1
2002	2,228	14.0
2003	2,116	15.9
2004	2,021	15.9
2005	1,874	17.1
2006	1,589	18.6
2007	1,805	19.2
2008	1,775	19.8
2009	1,913	22.4
2010	1,804	21.0
2011	1,748	23.6
Total	50,872	14.7

**Table 2:** Annual CSR (in percentage) by indications

Years	Breech presentation	CPD	Failure induction	Fetal distress	HIV positive	Malpresentation	Maternal disease	Placenta previa	Previous CS	Prolapsecord	Twins	Others
1992	1.95	3.18	0.21	1.74	0.00	0.08	0.24	0.26	3.24	0.11	0.08	0.24
1993	2.54	3.91	0.31	1.05	0.00	0.13	0.08	0.37	3.02	0.10	0.10	0.08
1994	1.75	3.92	0.25	0.93	0.00	0.25	0.31	0.42	3.78	0.00	0.20	0.11
1995	2.23	4.03	0.17	0.68	0.00	0.11	0.25	0.42	4.20	0.14	0.17	0.25
1996	2.10	3.99	0.37	1.08	0.00	0.18	0.26	0.50	4.14	0.16	0.13	0.26
1997	2.58	3.02	0.30	1.05	0.00	0.03	0.12	0.51	3.86	0.12	0.21	0.21
1998	2.04	3.35	0.79	1.35	0.00	0.07	0.31	0.45	4.10	0.14	0.17	0.10
1999	2.38	3.70	0.47	0.98	0.00	0.13	0.25	0.47	3.82	0.34	0.38	0.17
2000	2.75	3.66	0.25	0.58	0.00	0.25	0.25	0.58	4.03	0.29	0.29	0.37
2001	2.27	4.22	0.53	0.93	0.37	0.08	0.20	0.37	3.29	0.16	0.45	0.16
2002	2.78	3.99	0.22	0.94	0.54	0.04	0.31	0.67	3.46	0.22	0.45	0.22
2003	3.21	5.20	0.19	0.61	0.95	0.24	0.14	0.80	3.31	0.33	0.61	0.14
2004	2.87	5.34	0.35	1.24	0.79	0.30	0.15	0.69	3.32	0.15	0.25	0.25
2005	3.09	5.55	0.27	1.28	0.64	0.16	0.21	0.75	3.68	0.21	0.64	0.21
2006	3.34	5.41	0.57	1.57	0.82	0.31	0.31	0.63	4.15	0.06	0.69	0.19
2007	3.93	5.60	0.83	1.88	0.28	0.11	0.22	0.33	4.65	0.17	0.61	0.22
2008	3.27	6.25	0.45	1.24	0.06	0.28	0.23	0.56	5.75	0.17	0.73	0.23
2009	3.55	6.90	0.37	1.88	0.37	0.42	0.10	0.47	6.27	0.31	0.99	0.37
2010	3.10	6.21	0.22	1.66	0.28	0.33	0.33	0.72	6.32	0.11	0.72	0.39
2011	3.55	7.89	0.51	1.77	0.06	0.23	0.23	0.74	6.58	0.06	0.92	0.51
Total	2.61	4.47	0.36	1.18	0.20	0.17	0.22	0.51	4.09	0.16	0.37	0.22

During the study period, there were 2,720 pregnancies with previous cesarean section. Of them, 751 (27.6 %) had tried vaginal birth after cesarean section (VBAC) with a success rate of 75.9 % and 181 (24.1 %) needed a repeated cesarean section, because of failed VBAC.

## Discussion

Since the risks of medically unnecessary CSs outweigh the benefits, CSR is of most concern currently. In several countries, the CSR is urgently needed to be intensively controlled. Maternal mortality rate in CS is two to seven times higher than that in vaginal birth. In addition, morbidity rate is much higher, including more pain, longer and more difficult postpartum recovery, a higher likelihood of complications and cesarean sections in subsequent pregnancies, and more difficulty in conceiving after cesarean sections, as well as, greater likelihood of obstetric complications in subsequent pregnancies. Neonatal mortality and morbidity for unnecessary CS is much higher when compared to vaginal births. These are associated with higher prevalence of respiratory distress syndrome and persistent pulmonary hypertension in surviving newborns, and childhood asthma after CS compared to vaginal birth [18]. Several studies conclude that CS has a higher risk than vaginal delivery in many aspects [1, 8, 19], including maternal death, hysterectomy, bowel obstruction, surgical injuries, infections etc. Finally, unnecessary CSs are a huge waste of medical resources [2, 20]. The cost of the global saving by a reduction of CS rates to 15 % was estimated to be $2.32 billion (US dollars); the cost to attain a 10 % CS rate was $432 million (US dollars) [3]. Money spent on unnecessary CS must necessarily be taken away from money to fund necessary or desirable medical care for other medical conditions or for medically necessary cesareans that is unavailable for such reason [20]. Because of several reasons mentioned above, we are obligated to audit the CSR of our own.

CSR is markedly varied among institutions it should, therefore, be analyzed for its own rate. Though CSR has a tendency to increase all over the world, the extent of an increase is markedly different. For example, CSR in our public hospital as presented here has risen from 11 % in 1992 to 23.7 % in 2011, whereas, the CSR in a public hospital in Tehran, Iran, has increased from 14.3 % in 1979 to 85.3 % in 2009. Such a marked increase needs immediate strategies to prevent the rising trend and increasing number of unnecessary CS. To initiate effective strategies, the main indications responsible for such an increase must be determined, leading to correctly audit and they may be different from study to study.

However, overall CSR in our setting as a public hospital in developing country is not too high when compared to that reported in developed country, it is on the rise continuously. The rate in 2011 is twice that in 1992 and has a trend to further increase and possibly be as high as more than 80 % as reported in Tehran if immediate strategy is not adopted to prevent further rising. It should be noted that, CSR was relatively constant during the 10 year period before 2001 and the rate obviously increased during the last decade. Though overall CSR in our hospital (14.7 %) is relatively acceptable as WHO recommendations, the CSR in the recent year is too high and unacceptable. Though the CSR is increased 2-fold during the past two decades in our setting, it is relatively low compared to 85 % in public hospital in Tehran [17]. These findings imply that CSR in public hospitals be markedly varied from hospital to hospital and each country should has its own figures. Hopefully, this study may inspire other centers to analyze and audit their own CSRs.

Unlike most previous reports, this study not only focused on the rise in overall CSR over the time period, but it also analyzed for the extent of the rise in separate groups by indications for cesarean section. Interestingly, the primary causes of rising in CSR in our setting are mainly confined to indications of CPD and previous CS. These findings implied that the rising trend of CSR originated from over-diagnosis of CPD leading to unacceptable CSR. Immediate strategy should be adopted to prevent unnecessary CS in our setting and it should focus on a careful diagnosis of CPD. Hopefully, CSR due to previous CS would be decreased accordingly. Notably, while the threshold in diagnosis of CPD was lower in the recent years, resulting in a rise of CSR, cesarean section due to fetal distress is rather constant with a rate of 2 %, though it is significant higher than that in the past 20 years. The indication due to fetal distress is in an acceptable range. Though over-diagnosis of fetal distress significantly count on an increased CSR in several reports, it is only a minimal impact on CSR in our hospital.

CSR indicated by breech presentation has been obviously increased in the year of 2002 and then rather constant. In the year of 2011, breech presentation responsible for 3.5 % of all CSR, accounting for two-third of pregnancies with breech presentation in our hospital. Strategy to promote vaginal breech delivery should not be highly effective to prevent further rising CSR since the prevalence of breech presentation is only about 5–6 % of parturients. All attentions must be paid to diagnosis of CPD to prevent both primary and repeated CS. Other factors that might partly play a role in the rising trend included a higher proportion of elderly pregnant women and primigravid in the recent years. As already known, CSR is somewhat higher among these groups of women. However, changes in such proportions are out of control to achieve the optimal CSR.

Note that while the total number of deliveries had continuously declined during the study period, the CSR in all indications have been increased over such a period. Nevertheless, the increase is only modest in most indications except for CPD and previous CS. This study strongly suggests that intensive audit in diagnosis of CPD must be seriously taken into account to achieve the optimal CSR in public hospitals in our country. This should be a national consensus.

As mentioned above, each institution should assess its own CSR and associated indications since it may be different from others. For example, fetal distress had only minimal effect on the rising trend of CSR in our setting, while it greatly contributed in several studies. In one study, while 1.7 % of all birth were designated as involving fetal distress in 1980, 10 % were so in 1987 [7], and by 1989, 8.8 % were so designated [2]. To a large degree, this rise generally is a function of growing reliance upon EFM with high false positive rate [2], whereas, this is not the main problem in our hospital in which the rate of CS due to fetal distress is relatively stable around 2 % in the recent years.

Limitations in this study may include (1) several changes during such a long period of study such as improvement of hospital capacity and technology as well as changes in patient’s baseline characteristics. Certainly, these factors might have influenced on CSR, however, they were unlikely to change our conclusion. (2) It could not be assumed that our setting could be a representative of all public hospitals in Thailand and could not be a basis for national recommendations. However, our observations may encourage other hospitals to find out their own trends. However, practices in public sectors in our country are usually nearly the same. Therefore, though our settings might not represent our national status, our findings could suggest a trend in our country.

In conclusion, this study found that in our setting CSR has been gradually increased, especially in the last decades. The main reasons of such an increase are likely to be associated with over-diagnosis of CPD and subsequent repeated CS, while other indications play only a minimal role. To achieve the appropriate CSR, audit system for diagnosis of CPD must be instituted.
